# General Intelligence

**Published:** 1844-04

**Authors:** 


### GENERAL INTELLIGENCE.

*Surgical Patents.*—M. GANNAL, in Sept. 1837, took out a patent for a method of embalming, which consisted mainly in the injection of a solution of sulphate of alumina and of arsenic into the arterial system, and by virtue of this he maintained that he alone had the right in France to embalm by injection, and announced that he would prosecute any medical man who attempted to infringe his patent. M. Marchal, a distinguished young surgeon, conceiving this claim to be an infringement of the rights of the profession, publicly injected a body with a solution of arsenic, previously giving M. Gannal notice of his intended operation. M. Gannal, conceiving his patent to be infringed, brought a suit against M. Marchal before the "Tribunal Correctional" of Paris, and this tribunal have decided that the human body could not be assimilated to merchandize, and that it is not possible to take out a patent for any operation performed on it. The case was consequently discharged, and the costs, which are heavy, fall on M. Gannal.—*Medical News, March,* 1844.

---

*Fibrous tumours of the Mamma.*—Prof. CRUVEILHIER read to the French Academy of Medicine on the 9th of January last, a memoir on fibrous tumours of the breast, often mistaken for cancer. The conclusions to which the learned author has arrived are, 1st. That the mammæ may become the seat of fibrous tumours. 2d. That they are to be distinguished from scirrhus and cancer, from their not being adherent. 3d. That they never become cancerous, and ought not therefore to be extirpated.—*Ib.*

